# Universal Predictors of Dental Students’ Attitudes towards COVID-19 Vaccination: Machine Learning-Based Approach

**DOI:** 10.3390/vaccines9101158

**Published:** 2021-10-10

**Authors:** Abanoub Riad, Yi Huang, Huthaifa Abdulqader, Mariana Morgado, Silvi Domnori, Michal Koščík, José João Mendes, Miloslav Klugar, Elham Kateeb, IADS-SCORE

**Affiliations:** 1 Department of Public Health, Faculty of Medicine, Masaryk University, 625 00 Brno, Czech Republic; koscik@med.muni.cz (M.K.); klugar@med.muni.cz (M.K.); 2 International Association of Dental Students (IADS), 1216 Geneva, Switzerland; vpsr@iads-web.org (H.A.); mmorgado@egasmoniz.edu.pt (M.M.); vppr@iads-web.org (S.D.); 3 Czech National Centre for Evidence-Based Healthcare and Knowledge Translation (Cochrane Czech Republic, Czech EBHC: JBI Centre of Excellence, Masaryk University GRADE Centre), Institute of Biostatistics and Analyses, Faculty of Medicine, Masaryk University, Kamenice 5, 625 00 Brno, Czech Republic; 4 Department of Psychology, Faculty of Social Studies, Masaryk University, 602 00 Brno, Czech Republic; yihuang@mail.muni.cz; 5 Institute for Research of Children, Youth and Family, Faculty of Social Studies, Masaryk University, 602 00 Brno, Czech Republic; 6 Clinical Research Unit (CRU), Egas Moniz Cooperativa de Ensino Superior, 2829-511 Almada, Portugal; jmendes@egasmoniz.edu.pt; 7 Oral Health Research and Promotion Unit, Faculty of Dentistry, Al-Quds University, Jerusalem 510 00, Palestine; ekateeb@staff.alquds.edu; 8 Public Health Committee, World Dental Federation (FDI), 1216 Geneva, Switzerland

**Keywords:** COVID-19 vaccines, decision making, decision trees, dental education, international association of dental students, machine learning, mass vaccination, regression analysis

## Abstract

Background: young adults represent a critical target for mass-vaccination strategies of COVID-19 that aim to achieve herd immunity. Healthcare students, including dental students, are perceived as the upper echelon of health literacy; therefore, their health-related beliefs, attitudes and behaviors influence their peers and communities. The main aim of this study was to synthesize a data-driven model for the predictors of COVID-19 vaccine willingness among dental students. Methods: a secondary analysis of data extracted from a recently conducted multi-center and multi-national cross-sectional study of dental students’ attitudes towards COVID-19 vaccination in 22 countries was carried out utilizing decision tree and regression analyses. Based on previous literature, a proposed conceptual model was developed and tested through a machine learning approach to elicit factors related to dental students’ willingness to get the COVID-19 vaccine. Results: machine learning analysis suggested five important predictors of COVID-19 vaccination willingness among dental students globally, i.e., the economic level of the country where the student lives and studies, the individual’s trust of the pharmaceutical industry, the individual’s misconception of natural immunity, the individual’s belief of vaccines risk-benefit-ratio, and the individual’s attitudes toward novel vaccines. Conclusions: according to the socio-ecological theory, the country’s economic level was the only contextual predictor, while the rest were individual predictors. Future research is recommended to be designed in a longitudinal fashion to facilitate evaluating the proposed model. The interventions of controlling vaccine hesitancy among the youth population may benefit from improving their views of the risk-benefit ratio of COVID-19 vaccines. Moreover, healthcare students, including dental students, will likely benefit from increasing their awareness of immunization and infectious diseases through curricular amendments.

## 1. Introduction

The race towards achieving substantial levels of population immunity, commonly known as herd immunity, against the coronavirus disease (COVID-19) embraces a myriad of milestones that should be unlocked by the world’s governments by fall 2021 [1]. The immunization of young adults is one of these challenging milestones due to numerous reasons. First, the low risk of COVID-19 morbidity and mortality among the youth population can trigger reluctance and/or resistance to getting vaccinated against the severe acute respiratory syndrome coronavirus—2 (SARS-CoV-2) [2,3,4,5,6]. In a recent scoping review, Aw et al. 2021 found that the low self-perceived risk of contracting COVID-19 was an individual/group factor of COVID-19 vaccine hesitancy in high-income countries [7].

The COVID-19 mass vaccination strategies followed a priority approach that was suggested by the World Health Organization (WHO) and endorsed by all its member states; therefore, the older adults, healthcare personnel, essential workers, etc., were prioritized to receive COVID-19 vaccines at the expenses of the young adults who had to wait around six months until they were permitted to register for vaccination [8,9]. Mass media and social media play a key role in shaping youth views and attitudes towards health-related issues, including receiving vaccines [10,11,12,13,14,15]. The negative impacts of social media on vaccine acceptance levels were found in the Czech Republic, Palestine, the United Kingdom (UK), and the United States of America (US) [13,15,16,17].

University students are an interesting group for both public health research and interventions because they constitute a special subset of the general youth population that is supposed to retain the highest possible levels of health literacy [15,18,19,20]. Likewise, studying health-related subjects was found to be a pivotal promoter of health literacy; therefore, healthcare students represent the upper echelon of health literacy among the youth population [20]. The social role of healthcare students in health promotion is underscored by the hypothesis that those students are broadly perceived as opinion leaders within their local communities and social circles; therefore, the policies aiming to improve their health-related attitudes and behaviors can yield an indirect and long-term benefit for the public health literacy [21,22,23,24].

Dental students had been recruited in this study as representatives of healthcare students, even though they are challenged by additional strains during this pandemic that could have affected their willingness to accept the COVID-19 vaccine either positively or negatively [25,26,27,28,29]. The discontinuation of clinical training, the abrupt shift to online education, and the increased risk of contracting COVID-19 infection through aerosol-generating procedures were found to be affecting the dental students’ attitudes and behaviors amid the pandemic, which may promote their willingness to get vaccinated in order to overcome the pandemic restrictions [25,26,27,28,29]. Moreover, COVID-19 as a syndromic disease imposes further challenges to the dental practice through its puzzling oral manifestations, e.g., loss of taste (dysgeusia), oral ulcers, oral candidiasis, etc., that can complicate the timely and proper diagnosis of oral lesions [30,31,32,33,34,35,36,37]. Contrarily, dental students’ knowledge about the COVID-19 pandemic was barely adequate in some countries, especially the low-income ones, which might endanger their willingness to get vaccinated [38].

Prior to the COVID-19 pandemic, dental students were used to being vaccinated against various infections, including hepatitis B, tetanus, and influenza as part of their clinical training prerequisites [39]. In addition to high-income countries, the middle-income ones adopted intense policies during the last few years that yielded a significant increase in the percentage of immunized dentists [40,41]. Nonetheless, the attitudes of dental students toward novel vaccines, e.g., human papillomavirus (HPV) vaccine, were found to be unsatisfactory in the high and middle-income countries due to a lack of knowledge [42,43,44]. Recently, a growing number of studies had been designed to describe the attitudes of healthcare students towards the novel COVID-19 vaccines [11,13,15,45,46,47,48]. One of the major critiques of these studies is the lack of in-depth analysis of the promoters and barriers of vaccine willingness; therefore, it had been recommended to carry out a deep analysis utilizing machine learning-based approaches for evaluation of the predictors of COVID-19 vaccine-related attitudes [11].

Machine learning-based approaches had been widely utilized in understanding the epidemiology, clinical presentation, and outcomes of COVID-19, as well as its vaccine acceptance sentiments and vaccine side effects [49,50,51,52,53,54]. Artificial intelligence (AI) models could be as accurate as medical specialists in the diagnosis and prognosis of COVID-19; however, their current diagnostic accuracy needs to be improved through further integration of big datasets of radiographic and clinical information [49]. Discourse analyses of social media platforms, e.g., Twitter, Facebook, and YouTube, also utilized AI models to better understand COVID-19 vaccine misinformation and hesitancy [53,54,55]. Hussain et al. 2021 used AI to analyze Facebook and Twitter content in the UK and the US during March–November 2020, and their findings were highly correlated with the results of the concurrent national surveys in both countries [53]. Therefore, AI-enabled social media analysis was recommended for large-scale adoption by institutions and governments alongside survey-based techniques for real-time assessment of public sentiments of vaccination willingness [53].

The overall aim of this study was to synthesize a data-driven model for the predictors of COVID-19 vaccine willingness among dental students worldwide. The primary objective was to identify the important predictors of vaccination willingness from the pool of demographic and psychological independent variables, and the secondary objective was to articulate these predictors in a conceptual model following the socio-ecological theory.

## 2. Materials and Methods

### 2.1. Study Design

This analytical study is based on the data curated by an international cross-sectional survey carried out by the Standing Committee on Research and Education (SCORE) of the International Association of Dental Students (IADS) in February 2021 [56,57]. The survey aimed to evaluate the attitudes of dental students in twenty-two countries towards COVID-19 vaccination and to explore the potential drivers of their vaccine hesitancy stand [11]. A non-random sampling technique through snowballing recruitment (where the participants recruited other participants to fill in the questionnaire) was employed to collect data from the target population, and the digital form that curated data was coded and extended to the participating subjects using KoBoToolbox (Harvard Humanitarian Initiative, Cambridge, MA, USA, 2021) [58]. The national delegates of the IADS acted as liaison officers for data collection in their respective countries, and they used the communication platforms of their national students’ associations to promote the survey, e.g., mail lists, social media pages, and instant messaging groups [57].

The self-administered questionnaire (SAQ) used in this survey consisted of 20 multiple-choice items inquiring about a) demographic information (gender, age, academic level, and country), b) COVID-19-related experience (prior infection, caring for COVID-19 patients, or having a COVID-19 case or fatality within the social circle), c) willingness to receive COVID-19 vaccine (measured by a 5-point Likert scale), and d) the drivers of COVID-19 vaccine-related attitudes [11]. The SAQ was developed and validated through a panel of public health and medical education experts, then its test re-test reliability was established through a group of volunteer students that indicated that the English version of the used SAQ retained a perfect level of reliability. The whole validation process and psychometric properties of the SAQ had been described earlier somewhere [11].

The drivers of vaccine hesitancy evaluated in this study were adapted from the compendium of the Strategic Advisory Group of Experts on Immunization (SAGE) of the World Health Organization (WHO) [59]. Ten drivers were included, of which five were contextual (“influences arising due to historic, socio-cultural, environmental, health system/institutional, economic or political factors”), two were individual/group (“influences arising from personal perception of the vaccine or influences of the social/peer environment”), and three were vaccine-specific (“directly related to vaccine or vaccination”) drivers [60]. The impact of media and social media, celebrities and opinion leaders, trust in government, trust in the pharmaceutical industry, and cultural and religious values were deemed to be the contextual drivers in the SAQ, while the misconception of natural immunity and perceived knowledge were employed as the individual/group drivers. The vaccine-specific drivers were the risk/benefit ratio of COVID-19 vaccination, the attitudes towards new vaccines, and the local availability of the COVID-19 vaccine. (Table 1)

### 2.2. Study Variables

The downstream analyses utilized a higher version of the dataset that converted all the study variables into numerical variables. The target variable was the willingness to receive the COVID-19 vaccine, and its possible outcomes ranged between “Totally Disagree” denoted by 1 and “Totally Agree” denoted by 5. Vaccine acceptance was defined as the willingness to receive the COVID-19 vaccine once it becomes accessible, and it was denoted by the 4th and 5th levels of the Likert scale. Vaccine hesitancy was defined as the “delay in acceptance or refusal of vaccines despite availability of vaccinations services”, and it was denoted by the 3rd level of the Likert scale. Vaccine resistance was defined as the rejection of receiving vaccines despite of their local accessibility, and it was denoted by the 1st and 2nd levels of the Likert scale [61].

The predictor (candidate) variables included both demographic variables, COVID-19-related experience variables, and vaccine hesitancy drivers’ variables. The economic level variable was graded from low-income economies, denoted by 1, to high-income economies, denoted by 4. Each of the vaccine hesitancy drivers had three possible outcomes, “Yes” denoted by 2, “Not Sure” denoted by 1, and “No” denoted by 0 (Table 2).

### 2.3. Ethical Considerations

The study protocol was reviewed and approved by the Ethics Committee of the Faculty of Medicine, Masaryk University on 20 January 2021 under the Ref No. 4/2021. The cross-sectional survey was conducted in accordance with the Declaration of Helsinki for research involving human subjects, and it had been previously reported according to the Strengthening the Reporting of Observational Studies in Epidemiology (STROBE) guidelines for cross-sectional studies [11,62,63].

The study data was stored and processed in full compliance with the EU General Data Protection Regulation (GDPR); therefore, no identifying personal data was collected from the participants that might enable their retrospective identification [64]. Prior to participation in the survey, each participant had to give their informed consent digitally, and no information was saved if the participant quit the study at any stage before submitting their answers.

### 2.4. Data Mining

#### 2.4.1. Data Analysis

Initially, a regression decision tree analysis was carried out as the data mining approach to confirm the variables associated with vaccination willingness. Regression tree is a non-parametric technique that can find out the potential significant predictors for the target outcomes. After selection of the strongest candidate variables, we adopted a multi-level regression model based on the socio-ecological theory to predict individuals’ vaccination willingness by the candidate variables.

#### 2.4.2. Decision Tree Analysis

The R package of “rpart” was utilized to construct the regression decision tree by a machine learning manner. By using “sample function” in R, we divided the sample randomly into the training dataset, 70% of the whole sample and the testing dataset, 30% of the whole sample. For training the decision tree splits, we set the stopping rule of the minimum observed cases of a node at 30. Each split is sought to decrease the overall lack of fitness by a factor of 0.01 (cost complexity factor) before being attempted.

We examined the cross-validated errors, selected the complexity parameters associated with the minimum errors, and pruned them in order to avoid over fitting. Eventually, with the aim to estimate the validity of this trained decision tree, we computed the R^2^ of the linear regression relationship between observed values in the test dataset and predicted values by the trained decision tree.

#### 2.4.3. Multi-Level Regression

In our dataset, except for the economic level that was a contextual variable, all the other demographic variables belonged to the individual level, e.g., gender, academic level, clinical training, etc. The vaccine hesitancy drivers suggested by the SAGE also belonged to the individual level, e.g., trust in the government, trust in the pharmaceutical industry, and misconception of natural immunity, etc.

According to the socio-ecological theory, intrapersonal factors are nested at the contextual level [65]. Thus, if the decision tree decided that economic level was a significant predictor, the multi-level regression model would place the variable in level 2, and other candidate variables in level 1. This linear multi-level model was slope-fixed, as the relationships between the contextual factor and individual factors were fixed. We performed this step using the R-based open software Jamovi [66].

## 3. Results

### 3.1. Descriptive Statistics

A total of 6639 respondents were included in the downstream analyses, of which 70.5% were females and 63.5% were 17–22 years old. The most represented academic year was the third year (21.4%), followed by the fourth year (19%), and the second year (18.5%). According to the latest ranking of the World Bank, 74.4% of the participants were from upper-middle- and high-income countries [67].

Regarding their COVID-19-related experience, 16.6% had been previously infected by SARS-CoV-2, and 27.2% provided care to COVID-19 patients. While 87.4% of the participants knew someone who was infected by SARS-CoV-2, 45.7% of them knew someone who died due to SARS-CoV-2.

On answering the question of willingness to receive a vaccine against COVID-19, only 63.5% were vaccine accepting, 22.5% were vaccine hesitant, and 13.9% were vaccine resistant. About one-third (33.4%) of the participants acknowledged that their decision regarding vaccination was influenced by the reports they heard/read in the media/on social media, and 16.2% acknowledged that their vaccination decision was influenced by celebrities or religious or political leaders.

Interestingly, only 35.1% indicated their trust in their governments’ capacity to make appropriate decisions regarding the best vaccines to be provided, and 47.4% indicated their trust in the pharmaceutical industry to provide credible data on COVID-19 vaccines’ safety and effectiveness.

Over one quarter (26.4%) had the misconception of the superior effectiveness of natural immunity over vaccination against SARS-CoV-2, and only 31.5% felt that they had sufficient knowledge about COVID-19 vaccines and their safety. Almost one half (50.7%) of the participants thought that the benefits of COVID-19 vaccines outweighed their reported side effects and adverse reactions, and 43.2% exhibited positive attitude towards receiving novel vaccines generally. Regarding the local availability, 40.7% of the participants felt confident that their local health centers would have the COVID-19 vaccines whenever they need them (Table 3).

### 3.2. Decision Tree Analysis

**Figure 1:**
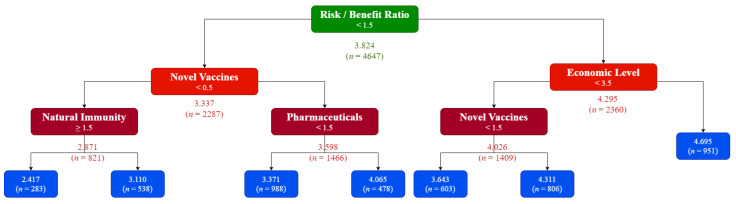
Decision tree for prediction of dental students’ willingness to receive COVID-19 vaccines, February 2021 (*n* = 6639). The values inside the colored blocks represent the cutoffs of the answers for each predictor when “No” = 0, “Not Sure” = 1, and “Yes” = 2. The values below the colored blocks represent the cutoff response to the COVID-19 vaccination willingness question when “Totally Disagree” = 1, “Disagree” = 2, “Not Sure” = 3, “Agree” = 4, and “Totally Agree” = 5.

On comparing the predicted values and observed values for the vaccination willingness in the test dataset based on a linear regression model, the R^2^ was 0.27, which means that the model can explain 27% of the variance of the dependent variable. The generated model suggested that there were five important predictors towards individuals’ vaccination willingness, those are (1) economic level of the country, (2) individual’s level of trust in the pharmaceutical industry, (3) individual’s misconception of natural immunity, (4) individual’s attitudes toward novel vaccines in general, and (5) individual’s views for the risk-benefit-ratio of vaccines against COVID-19 (Figure 1).

To interpret the generated model, the students should be primarily classified according to their views for the risk-benefit-ratio of COVID-19 vaccines. If they agreed that the benefits of the vaccines outweighed their reported side effects and adverse reactions, then only those from high-income countries would be very likely to accept the vaccine, but if they were from upper-middle-, lower-middle-, or low-income countries, their vaccination decision would be highly dependent on their attitudes toward novel vaccines.

If the students were not sure or disagreed that benefits of the vaccines outweighed their reported side effects and adverse reactions, then their attitudes toward novel vaccines would be a determinant factor. Those students who did not reject novel vaccines were to be classified according to their level of trust of the pharmaceutical industry in order to predict their willingness towards receiving COVID-19 vaccines, while those students who rejected novel vaccines were to be classified according to their misconception of natural immunity in order to predict their willingness towards receiving COVID-19 vaccines.

### 3.3. Bivariate Correlation

On running bivariate correlation analysis using Pearson’s correlation coefficient (*ρ*), the vaccination willingness was found to be significantly (*Sig.* < 0.001) associated with the five important predictors (country’s economic level, individual’s trust of pharmaceuticals, misconception of natural immunity, belief of risk-benefit-ratio, and attitudes towards novel vaccines).

The misconception of natural immunity was inversely correlated with all other predictors, as well as the vaccination willingness. It was poorly correlated with the economic level (*ρ* = −0.222; *Sig.* < 0.001), the trust of pharmaceutical industry (*ρ* = −0.113; *Sig.* < 0.001), the belief of risk-benefit-ratio (*ρ* = −0.125; *Sig.* < 0.001), the attitudes toward novel vaccines generally (*ρ* = −0.085; *Sig.* < 0.001), and the vaccination willingness (*ρ* = −0.267; *Sig.* < 0.001).

On the other hand, the economic level was directly correlated with all other predictors except for the misconception of natural immunity. The economic level was poorly correlated with the trust of the pharmaceutical industry (*ρ* = 0.209; *Sig.* < 0.001), the belief of the risk-benefit-ratio (*ρ* = 0.199; *Sig.* < 0.001), the attitudes toward novel vaccines generally (*ρ* = 0.167; *Sig.* < 0.001), and the vaccination willingness (*ρ* = 0.236; *Sig.* < 0.001).

While the trust of the pharmaceutical industry was poorly correlated with the economic level (*ρ* = 0.209; *Sig.* < 0.001), it was fairly correlated with the belief of the risk-benefit-ratio (*ρ* = 0.389; *Sig.* < 0.001), the attitudes toward novel vaccines generally (*ρ* = 0.396; *Sig.* < 0.001), and the vaccination willingness (*ρ* = 0.401; *Sig.* < 0.001). Similarly, the belief of the risk-benefit-ratio was poorly correlated with the economic level (*ρ* = 0.199; *Sig.* < 0.001), even though it was fairly correlated with the trust of pharmaceutical industry (*ρ* = 0.389; *Sig.* < 0.001), the attitudes towards novel vaccines generally (*ρ* = 0.385; *Sig.* < 0.001), and vaccination willingness (*ρ* = 0.390; *Sig.* < 0.001). 

The attitudes toward novel vaccines were poorly correlated with the economic level (*ρ* = 0.167; *Sig.* < 0.001), and they were fairly correlated with the trust of the pharmaceutical industry (*ρ* = 0.396; *Sig.* < 0.001), the belief of the risk-benefit-ratio (*ρ* = 0.385; *Sig.* < 0.001), and the vaccination willingness (*ρ* = 0.424; *Sig.* < 0.001) (Table 4).

### 3.4. Multi-Level Regression

On running multi-level regression analysis of the predictors, at level II where the slope was fixed, the variance of random intercepts of the economic level was 0.020, and the variance of the residuals was 1.039 Table 5.

At level I where the intercept was fixed, the trust of the pharmaceutical industry had a positive effect on vaccination willingness (*β* = 0.304; *Sig*. < 0.001). Likewise, the belief of the risk-benefit-ratio (*β* = 0.285; *Sig*. < 0.001) and the attitudes toward novel vaccines (*β* = 0.382; *Sig*. < 0.001) predicted the vaccination willingness positively and significantly. In contrast to the mentioned three variables at level I, the misconception of natural immunity in the same level showed a negative influence on vaccination willingness (*β* = −0.270; *Sig*. < 0.001) (Table 6).

### 3.5. Socio-Ecological Model

**Figure 2:**
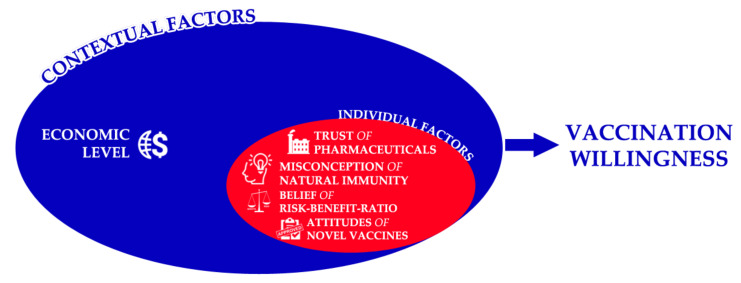
Conceptual map of vaccination willingness predictors according to the socio-ecological theory, February 2021 (*n* = 6639).

In accordance with the socio-ecological theory, the economic level of the country was controlled in level II as a contextual factor [68]. The correlation analysis results indicated that the higher the economic level where the students lived and studied predicted a higher propensity of vaccination willingness. The multi-level regression analysis suggested that in level I, the trust of the pharmaceutical industry, the belief that COVID-19 vaccine benefits outweighed their reported side effects and adverse reactions, and positive attitudes towards novel vaccines predicted vaccination willingness positively and significantly. The misconception of natural immunity decreased the vaccination willingness, and the regression model explained 30% of the variance (See Figure 2).

## 4. Discussion

In our study, a conceptual model was tailored based on the data of 6639 dental students worldwide who participated in a cross-sectional survey exploring their demographic characteristics, COVID-19-related experience, and vaccination willingness and its drivers. The model had been utilizing a machine learning-based approach to test and verify its components that indicated that there were five important predictors of COVID-19 vaccination willingness among dental students globally, i.e., the economic level, the trust of the pharmaceutical industry, the misconception of natural immunity, the belief of vaccines risk-benefit-ratio, and the attitudes toward novel vaccines. The socio-ecological theory had been employed to place the five predictors at two levels, i.e., the individual factors at level I and the contextual factors at level II.

The sole contextual factor suggested by our model was the economic level of the country where the students lived and studied. In agreement with this finding, Carrieri et al. 2021 synthesized a machine-learning model for vaccine hesitancy among Italian municipalities’ inhabitants, which indicated that socioeconomic indicators, e.g., the proportion of waste recycling and the employment rate, were the most powerful predictors of vaccine hesitancy at an area-level [69]. The household income was found to be a significant determinant for COVID-19 vaccine hesitancy among South African, Italian, and Portuguese adult populations during the pre-vaccination stage and the early stages of mass vaccination [70,71,72]. In a recent cross-sectional study, the lack of financial resources needed for health insurance was a trigger for COVID-19 vaccine hesitancy among US adults, thus emphasizing the role of a functional universal health coverage system in each country in the world, regardless of its economic level, to enhance the public’s vaccination willingness [73]. Similarly, Bertoncello et al. 2020 found that perceived financial hardship was a significant driver of parental vaccine hesitancy in Italy [74].

Trust of the pharmaceutical industry was one of the four individual predictors of COVID-19 vaccine willingness in our model. The recent cross-sectional studies of vaccine hesitancy found that mistrust of the pharmaceutical industry was very common among individuals with anti-vaccination positions in Austria, France, Italy, Malaysia, and the US [75,76,77,78,79]. Karafillakis et al. 2016 conducted a qualitative study for European healthcare workers, which indicated that in spite of their trust of their health systems, the mistrust of the pharmaceutical industry was profound among the participants, and it was suggested to be one of the promoters of vaccine hesitancy [80]. The European healthcare workers believed that pharmaceutical companies had financial interest that may retain them from disclosing safety and efficacy data of their products openly and place additional pressures on healthcare workers [80].

In their recent scoping review, Biswas et al. 2021 found that insufficient knowledge about the vaccines was a predictor for COVID-19 vaccine hesitancy among healthcare workers globally [81]. The misconception of natural immunity, which reflects the lack of factual knowledge about immunization, was the second individual predictor of vaccination willingness in our model. This misconception had been profoundly reported among the anti-vaccination parents, and it was dependent on their belief that illness is natural in childhood and the vaccine-preventable diseases are not life-threatening [82]. Therefore, this misconception can also be attributed to the low perceived risk of COVID-19 infection among young adults [3,4,5,6,7]. The general knowledge about vaccines had been consistently reported as a predictor of COVID-19 vaccination willingness, especially in low-income countries, e.g., Bangladesh, Cameroon, Ghana, Nigeria, and Palestine [83,84,85,86,87].

A nationwide survey of the French population indicated that the perceived risk–benefit balance (RBB) was a strong predictor for vaccination willingness among the surveyed parents [88]. The regression analysis confirmed that the perceived vaccine RBB was consistently unfavorable among the individuals with vaccine-hesitant and vaccine-resistant positions [88]. Another recent report had been jointly published by the WHO and the United Nations Children’s Fund (UNICEF) in 2017, and it revealed that the RBB was the most cited reason for vaccine hesitancy globally in the last few years [89]. In consistence with these results, our model depicted the RBB as the third individual predictor of COVID-19 vaccination willingness. The perceived vaccine RBB can be an emotional and intuitive process, rather than being based on logic and rationality, as the perceived risk of vaccination was commonly attributed to exaggerated feelings, e.g., the fear of potential adverse reactions, even the mildest ones [90]. Therefore, the independent (non-sponsored) research of post-vaccination side effects is believed to be a valid asset for delivering robust and non-biased evidence to hesitant individuals that can help them understand the nature of post-vaccination side effects [91,92,93,94,95,96,97,98,99]. The mild side effects, however non-life-threatening, can interfere with the daily routine of the vaccinated individuals and may require one or few days of absenteeism from work or school; therefore, they can be perceived as a vaccination barrier if the yielded benefit was not convincing [90].

The fourth individual predictor of vaccination willingness in our model was the general attitudes toward novel vaccines. The HPV vaccine is relatively novel, and the attitudes of dental students towards it were found to be unsatisfactory in the high- and middle-income countries due to the lack of knowledge [42,43,44]. The novel vaccines are usually associated with a unique set of concerns and fears, including the public apprehension over their safety and effectiveness; therefore, the WHO-SAGE issued guidelines in 2015 for controlling vaccine hesitancy, especially in the regions or the countries where new vaccines are introduced [100].

### 4.1. Strengths

Compared to the traditional studies, our research was innovatively based on the data mining technique, which is an efficient approach for analyzing big data, as there could be a mass of variables and interactions between each variable. Unlike most previous studies on the foundation of hypothesis-driven tests, the data mining-based method does not establish any initial assumptions. Traditional statistical methods aim to test the hypothesis extracted from previous evidences or theories by the criteria of type I error and type II error. In contrast, the data mining approach does not make any assumptions prior to analysis. The findings of this study are purely driven by data, while there were more than twenty COVID-19 vaccination drivers in our dataset. The machine learning-based methods can select the strongest predictors in one step, thus improving the analysis efficiency and simplifying the multi-level regression in the following step.

In addition, for a more robust explanation of results by data mining, we continued to build a multi-level regression model with the combination of the classical theory of socio-ecological framework, which helps researchers to understand the nested relationship of factors and avoid mixing all variables in one level simply. Moreover, the portion of explained variance by the multi-level regression (30%) had proven the accuracy of our decision tree model, which explained 27% of variance. Hence the more complicated multi-level regression that was based on the strong socio-ecological theory had an improved explanation of only 3%, which is much smaller when compared to the decision tree model (27%).

In a nutshell, our research not only adopted a data mining approach, which is an alternative way for multivariable analyses, especially for the complicated datasets, but also was rooted in a strong theory.

### 4.2. Limitations

The first limitation is that even though this study adopted a data mining strategy, we did not compare our results with other machine learning methods, e.g., network analysis. However, our decision tree model demonstrated high accuracy and validity with effective selection of significant predictors, even though other machine learning models have their unique advantages. For instance, the network analysis can visualize the strength of the associations of each candidate predictor and target variable. Our aim of using a machine learning-based approach was primarily to select the important predictors for the dependent variable, while we recommend further comparison of other varieties of machine learning-based approaches to interpret the dependent variables deeply based on pure data mining. The second limitation is that we could not perform longitudinal research to know participants’ real decision of COVID-19 vaccination in the future; it was impossible to compare our decision tree results with their final vaccination decision.

The third limitation is related to the non-random sampling technique that had been used to recruit the participants, as snowballing is prone to a number of biases that can affect the representativeness (external validity) of the recruited sample, e.g., self-selection bias. The fourth limitation is attributed to the social desirability bias, as the dental students may have tended to underreport their unfavorable attitudes/opinions of vaccination in order to look ideal and avoid criticism, even if they were assured that their answers will be completely anonymous.

### 4.3. Implications

This cross-sectional study-based model implies that longitudinal investigation of university students’ attitudes and decisions regarding COVID-19 vaccination is highly warranted to validate our proposed predictors. Based on the findings of our model, the interventions on the control of vaccine hesitancy among the youth population may benefit from improving their views of the risk-benefit ratio of COVID-19 vaccines through focusing on the societal benefits and being transparent about the potential side effects. Increasing the awareness of dental students, particularly, and healthcare students, generally, regarding vaccines will protect them from adopting misconceptions about immunization; such a target can be ideally achieved by amending the undergraduate dental curricula and giving more room for the infectious disease content.

## 5. Conclusions

In conclusion, the proposed conceptual model, which was based on the data of 6639 dental students worldwide, was developed through a machine learning-based approach to test and verify its components that indicated that there were five important predictors of COVID-19 vaccination willingness among dental students globally, i.e., the economic level of the country where the student lives and studies, the individual’s trust of the pharmaceutical industry, the individual’s misconception of natural immunity, the individual’s belief of vaccines risk-benefit-ratio, and the individual’s attitudes toward novel vaccines. According to the socio-ecological theory, the country’s economic level was the only contextual predictor, while the rest were individual predictors. Healthcare students, including dental students, will probably benefit from increasing their awareness of immunization and infectious diseases through curricular amendments.

## Figures and Tables

**Table 1:** The drivers of dental students’ COVID-19 vaccine hesitancy, February 2021 (*n* = 6639).

Item	Outcome
Contextual Drivers	
Do reports you hear/read in the media/on social media make you reconsider the choice to take the COVID-19 vaccine?	No
	Not Sure
	Yes
Do celebrities, religious or political leaders influence your decision about being vaccinated?	No
	Not Sure
	Yes
Do you trust that your government is making decisions in your best interest with respect to what vaccines are provided (e.g., your government purchases the highest quality vaccines available)?	No
	Not Sure
	Yes
Do you trust pharmaceutical companies to provide credible data on COVID-19 vaccine safety and the effectiveness of the vaccines?	No
	Not Sure
	Yes
Do you know anyone who will not take the vaccine because of religious or cultural values?	No
	Not Sure
	Yes
*If “Yes”, do you agree with these people?*	No
	Not Sure
	Yes
**Individual/Group Drivers**	
Do you think that there are better ways to prevent COVID-19 than using vaccines (e.g., developing immunity by becoming sick and recovering)?	No
	Not Sure
	Yes
Do you feel you have enough information about COVID-19 vaccines and their safety?	No
	Not Sure
	Yes
**Vaccine-Specific Drivers**	
Do you think that the benefits of COVID-19 vaccines outweigh their reported side effects/adverse reactions?	No
	Not Sure
	Yes
In general, when a new vaccine is introduced, are you inclined to consent to your vaccination?	No
	Not Sure
	Yes
Do you feel confident that the health center or doctor’s office will have the COVID-19 vaccines you need, when you need them?	No
	Not Sure
	Yes

**Table 2:** The enhanced dataset of dental students’ COVID-19 vaccine-related attitudes, February 2021 (*n* = 6639).

Variable	Outcome	Cd	Variable	Outcome	Cd	Variable	Outcome	Cd
Gender	Female	1	Knowing Fatality	No	0	Pharmaceuticals	Yes	2
	Male	2		Yes	1	Cultural Values	No	0
Academic Level	1st Year	1	Flu Vaccine	Never	0		Not Sure	1
	2nd Year	2		Sometimes	1		Yes	2
	3rd Year	3		Always	2	*Agreement* *with Values*	No	0
	4th Year	4		Mandatory	3		Not Sure	1
	5th Year	5	Willingness of Vaccination	Totally Disagree	1		Yes	2
	6th Year	6		Disagree	2	Natural Immunity	No	0
	Internship	7		Not Sure	3		Not Sure	1
	Fresh Graduate	8		Agree	4		Yes	2
Clinical Training	Pre-clinical	1		Totally Agree	5	Perceived Knowledge	No	0
	Clinical	2	Media/Social Media	No	0		Not Sure	1
Economic Level	Low income	1		Not Sure	1		Yes	2
	Lower-middle income	2		Yes	2	Risk/Benefit Ratio	No	0
	Upper-middle income	3	Public Figures	No	0		Not Sure	1
	High income	4		Not Sure	1		Yes	2
Prior Infection	No	0		Yes	2	Novel Vaccines	No	0
	Yes	1	Government	No	0		Not Sure	1
Providing Care	No	0		Not Sure	1		Yes	2
	Yes	1		Yes	2	Local Availability	No	0
Knowing Patient	No	0	Pharmaceuticals	No	0		Not Sure	1
	Yes	1		Not Sure	1		Yes	2

Cd = numerical code.

**Table 3:** Demographic characteristics, COVID-19-related experience, and COVID-19 vaccine willingness and its drivers among a global sample of dental students, February 2021 (*n* = 6639).

Variable	Outcome	Frequency (*n*)	Percentage (%)	Cumulative Percentage (%)
Demographic Characterstics				
Gender	Female	4682	70.5%	70.5%
	Male	1836	27.7%	98.2%
	Non-binary	53	0.8%	99%
	Prefer not to say	68	1%	100%
Academic Level	First Year	979	14.7%	14.7%
	Second Year	1227	18.5%	33.2%
	Third Year	1422	21.4%	54.6%
	Fourth Year	1259	19%	73.6%
	Fifth Year	817	12.3%	85.9%
	Sixth Year	240	3.6%	89.5%
	Internship/Fresh Graduate	695	10.5%	100%
Clinical Training	Pre-clinical Stage	2206	33.2%	33.2%
	Clinical Stage	4433	66.8%	100%
Economic Level	Low-income Country	467	7%	7%
	Lowe-middle-income Country	1232	18.6%	25.6%
	Upper-middle-income Country	3035	45.7%	71.3%
	High-income Country	1905	28.7%	100%
COVID-19-Related Experience				
Prior Infection	Yes	1105	16.6%	16.6%
	No	5534	83.4%	100%
Providing Care	Yes	1808	27.2%	27.2%
	No	4831	72.8%	100%
Knowing Patient	Yes	5801	87.4%	87.4%
	No	838	12.6%	100%
Knowing Dead	Yes	3031	45.7%	45.7%
	No	3608	54.3%	100%
Attitudes Towards COVID-19 Vaccine				
I am willing to take the COVID-19 vaccine once it becomes available to me.	Totally Disagree	491	7.4%	7.4%
	Disagree	434	6.5%	13.9%
	Not Sure	1494	22.5%	36.4%
	Agree	1495	22.5%	59%
	Totally Agree	2725	41%	100%
Contextual Drivers				
Do reports you hear/read in the media/on social media make you re-consider the choice to take the COVID-19 vaccine?	No	2903	43.7%	43.7%
	Not Sure	1519	22.9%	66.6%
	Yes	2217	33.4%	100%
Do celebrities, religious or political leaders influence your decision about being vaccinated?	No	4734	71.3%	71.3%
	Not Sure	827	12.5%	83.8%
	Yes	1078	16.2%	100%
Do you trust that your government is making decisions in your best interest with respect to what vaccines are provided?	No	2178	32.8%	32.8%
	Not Sure	2130	32.1%	64.9%
	Yes	2331	35.1%	100%
Do you trust pharmaceutical companies to provide credible data on COVID-19 vaccine safety and the effectiveness of the vaccines?	No	1448	21.8%	21.8%
	Not Sure	2041	30.7%	52.6%
	Yes	3150	47.4%	100%
Do you know anyone who will not take the vaccine because of religious or cultural values?	No	4286	64.6%	64.6%
	Not Sure	830	12.5%	77.1%
	Yes	1523	22.9%	100%
*If “Yes”, do you agree with these people?*	No	6260	94.3%	94.3%
	Not Sure	192	2.9%	97.2%
	Yes	187	2.8%	100%
Individual/Group Drivers				
Do you think that there are better ways to prevent COVID-19 than using vaccines?	No	2928	44.1%	44.1%
	Not Sure	1955	29.4%	73.6%
	Yes	1756	26.4%	100%
Do you feel you have enough information about COVID-19 vaccines and their safety?	No	2710	40.8%	40.8%
	Not Sure	1838	27.7%	68.5%
	Yes	2091	31.5%	100%
Vaccine-Specific Drivers				
Do you think that the benefits of COVID-19 vaccines outweigh their reported side effects/adverse reactions?	No	1188	17.9%	17.9%
	Not Sure	2082	31.4%	49.3%
	Yes	3369	50.7%	100%
In general, when a new vaccine is introduced, are you inclined to consent to your vaccination?	No	1606	24.2%	24.2%
	Not Sure	2162	32.6%	56.8%
	Yes	2871	43.2%	100%
Do you feel confident that the health center or doctor’s office will have the COVID-19 vaccines you need, when you need them?	No	1778	26.8%	26.8%
	Not Sure	2158	32.5%	59.3%
	Yes	2703	40.7%	100%

**Table 4:** Bivariate correlation among COVID-19 vaccine willingness and its suggested predictors, February 2021 (*n* = 6639).

		Economic Level	Pharmaceutical Industry	Natural Immunity	Risk/Benefit Ratio	Novel Vaccines	Vaccination Willingness
**Economic Level**	*ρ*	1.000					
	*Sig.*	*N/A*					
**Pharmaceutical Industry**	*ρ*	0.209	1.000				
	*Sig.*	<0.001	*N/A*				
**Natural Immunity**	*ρ*	−0.222	−0.113	1.000			
	*Sig.*	<0.001	<0.001	*N/A*			
**Risk/Benefit Ratio**	*ρ*	0.199	0.389	−0.125	1.000		
	*Sig.*	<0.001	<0.001	<0.001	*N/A*		
**Novel Vaccines**	*ρ*	0.167	0.396	−0.085	0.385	1.000	
	*Sig.*	<0.001	<0.001	<0.001	<0.001	*N/A*	
**Vaccination Willingness**	*ρ*	0.236	0.401	−0.267	0.390	0.424	1.000
	*Sig.*	<0.001	<0.001	<0.001	<0.001	<0.001	*N/A*

Pearson bivariate correlation analysis was carried out with a significance level (*Sig.*) < 0.05, and the Pearson’s coefficient was rho (*ρ*).

**Table 5:** Random components of the predictors of willingness to receive COVID-19 vaccines, February 2021 (*n* = 6639).

Groups	Name	SD	Variance	ICC
**Economic Level**	(Intercept)	0.142	0.0202	0.0191
**Residual**		1.019	1.0388	

Multi-level linear regression analysis was carried out. ICC refers to the interclass correlation coefficient, which is used to describe the portion of explained variance by random effects.

**Table 6:** Fixed effects parameters estimates of the predictors of willingness to receive COVID-19 vaccines, February 2021 (*n* = 6639).

			95% Confidence Intervale				
Predictor	Estimate	SE	Lower	Upper	df	*t*	*Sig.*
**(Intercept)**	3.809	0.073	3.666	3.951	2.96	52.3	<0.001
**Pharmaceutical Industry**	0.304	0.018	0.269	0.340	6633.72	16.8	<0.001
**Natural Immunity**	−0.270	0.016	−0.301	−0.239	6604.25	−17.1	<0.001
**Risk/Benefit Ratio**	0.285	0.019	0.248	0.322	6633.77	15.2	<0.001
**Novel Vaccines**	0.382	0.018	0.347	0.417	6633.99	21.5	<0.001

Multi-level linear regression analysis was carried out with a significance level (*Sig.*) < 0.05.

## Data Availability

The data that support the findings of this study are available from the corresponding author upon reasonable request.

## Appendix A. Members of IADS-SCORE Consortium

- Faculty of Dentistry, McGill University, Canada (Jacques Jaar); Schulich School of Medicine & Dentistry, Western University, Canada (Nima Lighvan); Faculty of Dentistry, University of British Columbia, Canada (Karen Lin);
- Faculty of Dentistry, SEGi University, Malaysia (Sandy Tan Qing Wen); Faculty of Dentistry, University of Malaya, Malaysia (Tan Hian Wei);
- School of Dentistry, Kathmandu University School of Medical Sciences, Nepal (Nitesh Singh);
- Department of Oral and Maxillofacial Surgery, School of Dentistry, Zanjan University of Medical Sciences, Iran (Parsa Firoozi); Department of Oral and Maxillofacial Surgery, School of Dentistry, Islamic Azad University Isfahan, Iran (Mohammad Mostafa Aghamohseni; Samin Sirous);
- Institute of Dentistry, CMH Lahore Medical College and Institute of Dentistry, Pakistan (Aneeqa Aslam; Maha Sohail); Dental College, Akhtar Saeed Medical and Dental College, Pakistan (Mehroz Ahmad Khan);
- Faculty of Dentistry, Beirut Arab University, Lebanon (Julien Issa; Mirna Abou Ibrahim);
- Faculty of Dental Medicine, Catholic University of Portugal, Portugal (António Coimbra Amaral);
- Faculty of Dental Sciences, Aldent University, Albania (Ersid Domnori);
- Faculty of Odontology, Universidad San Francisco De Quito, Ecuador (Jorge Ayala); Faculty of Odontology, Catholic University of Cuenca, Ecuador (Maria Sol Medina);
- Faculty of Dentistry, Universitas Indonesia, Indonesia (Viandra Tjokroadiredjo; Farih Aminah)
- Department of Dentistry, Al-rafidain University College, Iraq (Noor Sarmad); College of Dentistry, Uruk University, Iraq (Nabaa Abduladheem); Department of Dentistry, Al-rasheed University College, Iraq (Batool Mohammed);
- Department of Neuroscience, Reproductive Sciences and Dentistry, University of Naples Federico II, Italy (Matteo Cafasso); Department of Biomedical, Surgical and Dental Sciences, School of Dentistry, University of Milan, Italy (Gregorio Tortora); Department of Medicine and Surgery, School of Dentistry, University of Insubria, Italy (Anita Homayuni);
- Faculty of Dentistry, Riga Stradiņš University, Latvia (Kristīne Romanovska);
- Faculty of Odontology, Medical Academy, Lithuanian University of Health Sciences, Lithuania (Kriste Trijonytė; Julius Mikonis);
- Faculty of Dentistry, University of Khartoum, Sudan (Ahmed Abdalla); Faculty of Dentistry, Al Neelain University, Sudan (Zeinab Hassan); Faculty of Dentistry, University of Medical Sciences and Technology, Sudan (Aya Abdelrahim);
- Faculty of Dental Medicine, Monastir University, Tunisia (Haythem Ben Hadj Belgacem; Maya Fedhila);
- Faculty of Dentistry, Istanbul University, Turkey (İrem Erdoğdu); Faculty of Dentistry, Beykent University, Turkey (Berk Koparan); Faculty of Dentistry, Yeditepe University, Turkey (Ezgi Y eşiltan); Faculty of Dentistry, Marmara University, Turkey (Serap Beşiroğlu);
- Faculty of Dentistry, Ural State Medical University, Russia (Tatiana Spitsyna);
- Faculty of Dental Medicine, University of Rijeka, Croatia (Valentina Marasović; Elizabeta Vrkljan; Lovre Labura);
- Institute of Dentistry, Faculty of Medicine, University of Tartu, Estonia (Estelle Saavaste);
- College of Dentistry, University of Florida, United States of America (Natalie Atyeo); School of Dentistry, University of Michigan, United States of America (Alexandra Herzog);
- Oral Health Research and Promotion Unit, Faculty of Dentistry, Al-Quds University, Palestine (Mayar Danadneh)
